# Expression profiling of *Crambe abyssinica *under arsenate stress identifies genes and gene networks involved in arsenic metabolism and detoxification

**DOI:** 10.1186/1471-2229-10-108

**Published:** 2010-06-14

**Authors:** Bibin Paulose, Suganthi Kandasamy, Om Parkash Dhankher

**Affiliations:** 1 Department of Plant, Soil, and Insect Sciences, University of Massachusetts, Amherst, MA 01002, USA; 2 Undergraduate Student, School of Arts and Sciences, Cornell University, Ithaca, NY 14853, USA

## Abstract

**Background:**

Arsenic contamination is widespread throughout the world and this toxic metalloid is known to cause cancers of organs such as liver, kidney, skin, and lung in human. In spite of a recent surge in arsenic related studies, we are still far from a comprehensive understanding of arsenic uptake, detoxification, and sequestration in plants. *Crambe abyssinica*, commonly known as 'abyssinian mustard', is a non-food, high biomass oil seed crop that is naturally tolerant to heavy metals. Moreover, it accumulates significantly higher levels of arsenic as compared to other species of the Brassicaceae family. Thus, *C. abyssinica *has great potential to be utilized as an ideal inedible crop for phytoremediation of heavy metals and metalloids. However, the mechanism of arsenic metabolism in higher plants, including *C. abyssinica*, remains elusive.

**Results:**

To identify the differentially expressed transcripts and the pathways involved in arsenic metabolism and detoxification, *C. abyssinica *plants were subjected to arsenate stress and a PCR-Select Suppression Subtraction Hybridization (SSH) approach was employed. A total of 105 differentially expressed subtracted cDNAs were sequenced which were found to represent 38 genes. Those genes encode proteins functioning as antioxidants, metal transporters, reductases, enzymes involved in the protein degradation pathway, and several novel uncharacterized proteins. The transcripts corresponding to the subtracted cDNAs showed strong upregulation by arsenate stress as confirmed by the semi-quantitative RT-PCR.

**Conclusions:**

Our study revealed novel insights into the plant defense mechanisms and the regulation of genes and gene networks in response to arsenate toxicity. The differential expression of transcripts encoding glutathione-S-transferases, antioxidants, sulfur metabolism, heat-shock proteins, metal transporters, and enzymes in the ubiquitination pathway of protein degradation as well as several unknown novel proteins serve as molecular evidence for the physiological responses to arsenate stress in plants. Additionally, many of these cDNA clones showing strong upregulation due to arsenate stress could be used as valuable markers. Further characterization of these differentially expressed genes would be useful to develop novel strategies for efficient phytoremediation as well as for engineering arsenic tolerant crops with reduced arsenic translocation to the edible parts of plants.

## Background

Arsenic (As) is ubiquitous in the earth's crust and has widely been known as an acute poison and carcinogen [1,2]. Chronic As ingestion from contaminated food supplies and drinking water is strongly associated with an increased risk of liver, kidney, skin, and lung cancer [1]. Inorganic forms of arsenic, pentavalent arsenate (AsV) and trivalent arsenite (AsIII), are more toxic than the organic forms and are highly abundant in the environment. High levels of As in soil and drinking water have been reported around the world including Bangladesh, India, USA, and South America, where As in drinking water exceeds 10 ppb, the permissible limit established by the World Health Organization [3]. Industrial and agricultural practices, including the use of As in pesticides and herbicides [4], wood preservatives [5], and irrigation with contaminated groundwater [6,7], have significantly increased As levels in agricultural soils. Several studies indicated that food crops such as rice and vegetables grown on As contaminated soil can accumulate high levels of As in roots, shoots and seeds [7-9]. Thus As uptake by crop plants plays an important role in transfer of this toxic element into the food chain. Additionally, inorganic As species are phytotoxic and the elevated concentration of As in the soil causes a significant reduction in crop yield [7,10,11]. Therefore, developing strategies for remediation of As contaminated agricultural soil and water and decreasing the As contents in the food crops for preventing As contamination of the food chain are highly desirable. Furthermore, the phytotoxic effects suffered by crops grown in soil with As residues could be overcome by developing crops resistant to As. Progress towards developing such genetics-based strategies, however, has been hindered by the lack of a basic understanding of the molecular and biochemical mechanisms of As uptake and detoxification in higher plants.

Recently, plants have been engineered to detoxify and accumulate As in the aboveground tissues. Dhankher et al [12] engineered Arabidopsis with bacterial arsenate reductase, ArsC, and γ-glutamylecysteine synthatase, γ-ECS, thereby increasing the As accumulation and tolerance. This innovative study showed that the pathways for As metabolism could be engineered in plants either for hyperacumulation and tolerance or preventing the uptake of As in the food chain once we better understand the details of As metabolism in plants. While the molecular mechanism of As detoxification and tolerance has yet to be fully determined in plants, it has been shown that plants detoxify As by reducing AsV to AsIII, which is subsequently detoxified via forming complexes with thiol-reactive peptides such as γ-glutamylcysteine (γ-EC), glutathione (GSH), and phytochelatins (PCs) [13-16]. The AsIII-thiol complexes are suggested to be sequestered into vacuoles by glutathione-conjugating pumps, GCPs, although this still remains to be proven.

Recently, an arsenate reductase in Arabidopsis (AtACR2), which is a CDC25 phosphatase homologue, has been isolated and characterized [17,18]. The RNAi-mediated silencing of AtACR2 increased the sensitivity of Arabidopsis to AsV but not to AsIII, and the ACR2-deficient Arabidopsis plants translocated significantly higher amount of As in the aboveground tissues as compared to wild type plants [17]. Arsenate reductases have also been isolated from *P. vitatta *and rice that complemented the AsV sensitivity of yeast mutants deficient in arsenate reductase, ACR2 [19,20]. In addition, a glutaredoxin, PvGrx5, has been reported to have a role in regulating the intracellular levels of AsIII in *P. vittata *[21].

A comparative transcriptome analysis of AsV and AsIII stress in rice seedlings showed differential expression of a wide array of transcripts encoding transporters, regulatory genes, and genes involved in various stress responses [22]. Recently, two silicon transporters, LsiI and LsiII, which belong to the nodulin 26-like intrinsic proteins (NIPs) subfamily of the plant aquaporins, have been shown to co-transport AsIII and thus playing a major role in the entry of AsIII into rice roots [23]. Other members of NIP subfamily from Arabidopsis, rice, and lotus have been shown to facilitate the bidirectional transport of AsIII in yeast lacking the endogenous aquaglyceroporin transporter, FPS1 [24].

Several plant species, especially members of Brassicaseae and pteridophytes such as *P. vittata*, have been identified as promising candidates for phytoremediation [25,26]. In spite of all the recent As-related studies, we are still far from a comprehensive understanding of As uptake, detoxification, and sequestration in plants including *P. vittata*. Therefore, a better understanding of the molecular and biochemical mechanism of As uptake and detoxification in plants is needed to develop cost-effective strategies for phytoremediation as well as for engineering of As resistant food crops with reduced As accumulation. *C. abyssinica*, a member of Brassicaceae commonly known as 'abyssinian mustard', is a non-food, high biomass crop with short life cycle that holds potential for efficient phytoremediation of arsenic contaminated soils and sediments. *C. abysinnica*, referred to here as crambe, can be grown wherever oilseed rape is grown and it does not out-cross with canola or any other agriculture crop [27,28]. Moreover, crambe is highly resistant to As and other heavy metals, and accumulates significantly higher levels of As as compared to other Brassica species [29]. For these reasons, we selected crambe to study the molecular mechanism of As accumulation and detoxification and identify genes that are differentially regulated by AsV exposure. In this study, we isolated differentially expressed cDNAs from crambe exposed to AsV employing the PCR-Select Suppression Subtraction Hybridization (SSH) approach. We report here many uncharacterized novel transcripts as well as those participating in known biochemical pathways that are differentially expressed due to arsenate exposure.

## Results and Discussion

### Arsenic accumulation and tolerance in crambe

**Figure 1:**
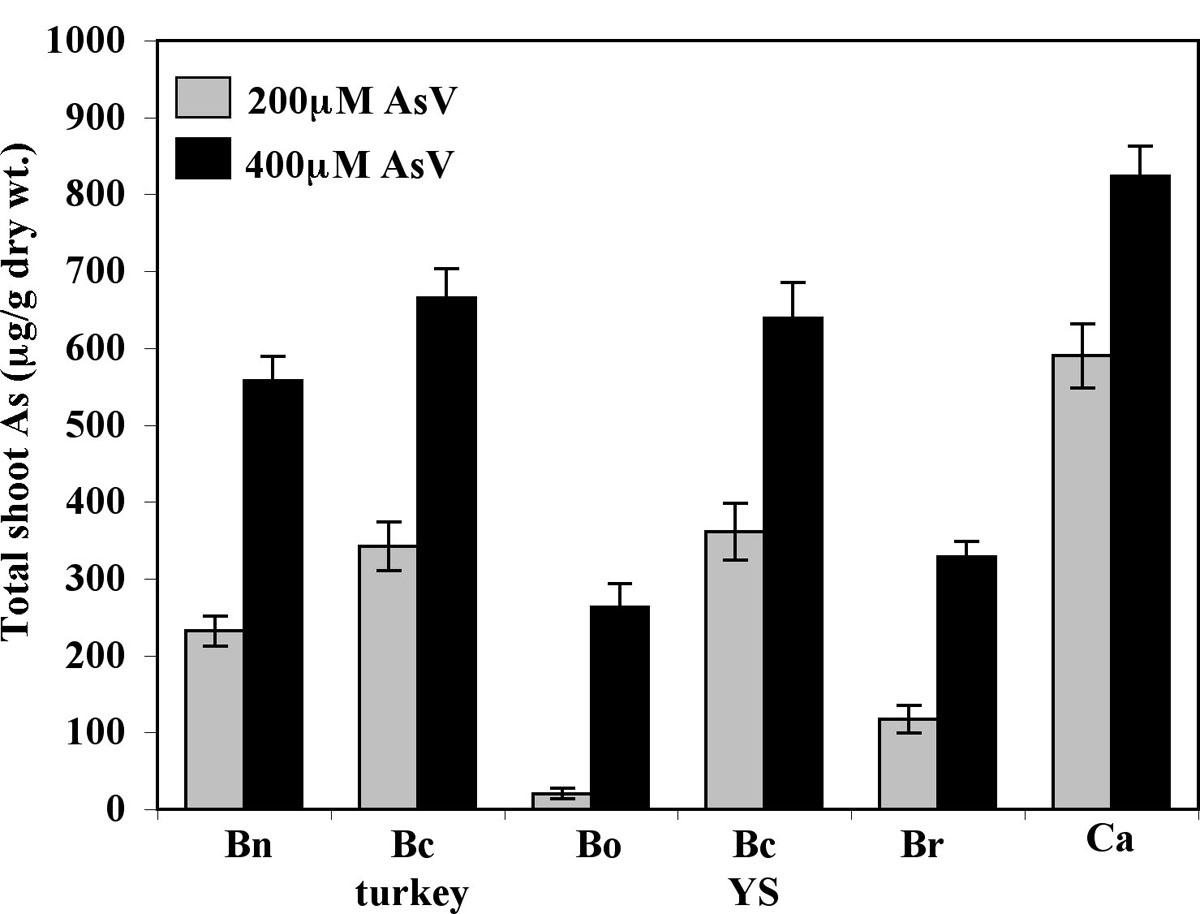
**Arsenic accumulation in *C. abyssinica *and other Brassica species seedlings grown in MS medium with different concentrations of As**. Total As contents in shoot tissues of *C. abyssinica *(Ca) compared with *Brassica napus *(Bn), *B. compestris *cv Turkey (Bc Turkey), *B. compestris *cv yellow sarson (Bc YS), *B. oleracea *(Bo) and *B. rapa *(Br).

**Figure 2:**
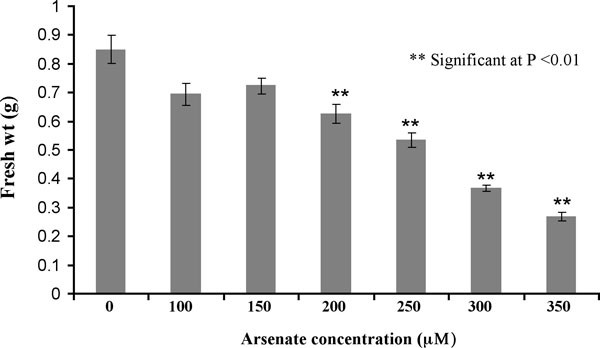
**Effect of various concentrations of arsenate on fresh biomass accumulation in *C. abyssinica *seedlings**. Fresh weight shown here is the average of 20 seedlings.

In order to select a plant suitable for studying As tolerance, we compared As accumulation in crambe plants to that of five other Brassica family members, namely *Brassica napus*, *B. compestris *cv Turkey, *B. compestris *cv Yellow Sarson, *B. oleracea *and *B. rapa*. Crambe plants were more tolerant to As than the various Brassica species examined and accumulated significantly higher amounts of As in aboveground shoot tissues followed by *B. compestris *and *B. rapa *(Figure 1). Similar results have been shown for uptake of As and Cd in crambe [29]. This suggests that crambe has an inherent capacity to accumulate As and other toxic metals at higher levels than other Brassica species. Thus, crambe plants are highly suitable for examining AsV stress response. To determine the optimum concentration of AsV required for inducing plant response, we treated 10 days old crambe seedlings with varying concentrations of sodium arsenate (0 to 350 μM) for seven days. The fresh weight of plants decreased moderately at 200 and 250 μM sodium arsenate concentrations with no symptoms of severe cellular damage (Figure 2). At higher concentrations (300 and 350 μM), however, AsV was toxic to plants as they were severely stunted with highly reduced biomass and had visible injury symptoms on leaves. Low levels of AsV (100 and 150 μM) have little effect on fresh weight or plant morphology. Therefore, an effective concentration of 250 μM sodium arsenate was selected to treat crambe seedlings to induce AsV stress.

### Differentially expressed crambe transcripts in response to arsenate stress

**Figure 3:**
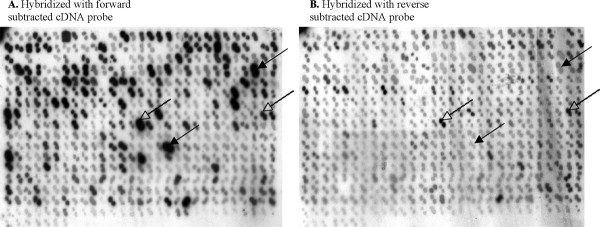
**Colony Array for differential screening of subtracted cDNA clones**. Membranes containing colonies expressing subtracted cDNAs hybridized with ^32^P-labeled forward subtracted probe (**A**) and reverse subtracted probe (**B**). Dotted arrows indicate false positives and solid arrows indicate positive colonies.

We used a PCR-Select SSH approach to isolate the differentially expressed transcripts from crambe in response to AsV exposure. As the ratio of driver to tester affects the stringency of the subtraction, two parallel subtractions were performed varying the driver/tester ratio and thus a total of 435 subtracted cDNA clones were obtained. A differential screening procedure finally identified 105 clones containing the differentially expressed mRNA sequences eliminating a large number of false positives as visible on the blots (Figure 3). We subjected all 105 subtracted cDNA sequences to homology search against the NCBI nucleotide database and found that they represented 38 different coding transcripts (Table 1). These crambe cDNA sequences were highly similar to that of Arabidopsis, as both species belong to the Brassicaceae family. A number of transcripts in our subtracted cDNA library were homologous to well characterized proteins, however several transcripts encode uncharacterized proteins that could be involved in novel pathways of As metabolism in plants.

**Table 1:** Relative frequency, identity, and description of differentially expressed subtracted cDNA transcripts from *C. abyssinica *after 24 hrs exposure to arsenate

Transcripts relative frequency(%)	Description	AGI locus ID	E value	Max. Identity (%)
5	Peptide methionine sulfoxide reductase (PMSR)	At5g07470	9.00E-85	80
4	Aldo/keto reductase (AKR)	At1g10810	4.00E-51	75
1	Serine palmitoyl transferase (SPT)	At3g48780	0.00E+00	89
5	Oxophyto dienoate reductase (OPR)	At1g76680	5.00E-132	90
12	Glutathione-S-Transferase *Tau *subfamily (GST-Tau)	At2g29460	3.00E-149	86
4	Glutathione S Transferase *Phi *subfamily (GST-Phi)	At4g02520	0.00E+00	89
1	Monodehydro ascorbate reductase (MDAR)	At3g52880	0.00E+00	92
4	Adenosine phosphosulfate kinase (APSK)	At2g14750	0.00E+00	87
1	Sulfate adenylyltransferase (APS)	At4g14680	8.00E-176	89
1	Sulfite reductase (SiR)	At5g04590	6.00E-106	92
1	20S proteasome beta subunit (20SPBS)	At1g56450	2.00E-164	87
4	Uubiquitin 14 (UBQ 14)	At4g02890	9.00E-91	87
3	Ubiquitin-associated (UBA)/TS-N domain- containing protein (Ubq.assoc.)	At4g24690	8.00E-105	82
1	MATE - family drug transporter (MATE)	At1g33110	3.00E-82	85
1	ChaC like protein (ChaC)	At5g26220	7.00E-69	80
1	Unknown (UK17)	At3g19990	8.00E-118	91
1	Unknown (UK231)	At5g08060	1.00E-50	75
1	Unknown (UK325)	At5g14260	2.00E-94	78
1	Unknown (UK21)	At5g61820	1.00E-122	79
1	Unkown similar to luminal binding protein (UK lum)	At5g42020	0.00E+00	92
1	Fructose bi-phosphate aldolase (FBPA)	At2g36460	5.00E-113	87
3	Glyceraldehyde 3 phosphate dehydrogenase (GAPDH)	At3g04120	5.00E-171	94
1	Aspartate amino transferase (AAT)	At4g31990	1.00E-94	90
3	Defense related protein (DRP)	At4g30530	1.00E-146	86
1	Trypsin inhibitor (Tryp.Inb)	At1g73260	2.00E-119	78
1	Pathogenesis related protein (PRP)	At4g33720	5.00E-88	86
1	Tryptophan synthase (Trp.syn)	At3g54640	0.00E+00	89
1	Thioglucosidase precursor (Thiogluc.)	At3g09260	1.00E 89	87
12	Iron ion binding oxidoreductase (FeBOx)	At3g19000	1.00E-174	84
1	RNA-binding domain containing protein (RNA bind.)	At5g09880	2.00E-118	85
1	Cystein proteinase (Cys.pro)	At4g16190	4.00E-30	76
1	Aldose 1 Epimerase (A1E)	At4g25900	1.00E-160	83
15	Heat shock protein (HSP)	At4g27670	1.00E-149	81
1	Beta tubulin (β- tub.)	At5g44340	0.00E+00	91
1	Male sterility family protein (MS5)	At5g48850	0.00E+00	91
1	Anthranilate benzoyl transferase (ABT)	At5g48930	3.00E-157	83
1	Transport protein particle componentBet3plike (TRAPP)	At5g54750	0.00E+00	90
1	Extracellular dermal glycoprotein (EDGP)	At1g03220	1.00E-76	85

The identity of the subtracted cDNAs suggested that AsV stress significantly affects pathways related to oxidative stress, defense, ion transport, sulfur assimilation, signal transduction, and photosynthesis and metabolism. Among the subtracted cDNA sequences (Table 1), glutathione-S-transferase (GSTs, both *tau *and *phi *subfamilies) was represented by the maximum number of clones (16%) followed by the heat shock proteins (15%), various reductases (15%), and proteins in the ubiquitin pathway (8%) indicating the significance of AsV as an acute cytotoxin. An iron-binding oxidoreductase (FeBOx) that participates in phenylpropanoid metabolism and has a functional domain similar to that of anthocyanidin synthase [30] represented 12% of cDNA clones in the library. Most of the remaining sequences encode proteins involved in various metabolic pathways including sulfate assimilation, carbohydrate and nitrogen metabolism, and amino acid synthesis. AsV stress also induced several general stress and defense-related proteins. Transcripts that code for a cation transporter associated protein, ChaC, and a secondary multidrug and toxin extrusion pump (MATE) along with many proteins of unknown function were also present in the library.

An arsenic-induced transcription profile has recently been reported in Arabidopsis [31] and rice [22] and identified GSTs as highly represented proteins in response to AsV exposure, followed by antioxidant synthesis and proteins involved in sulfur transport and metabolism. Our results for the transcriptional response of crambe to AsV support this consensus. In addition, our library contained the transcripts encoding glucanases, RNA binding proteins, proteins involved in amino acid biosynthesis as reported in Arabidopsis [31], as well as HSPs, MATE, glucosidases, jasmonic acid synthesis, and glyceraldehyde 3-phosphate dehydrogenase (GAPDH) as reported in rice [22,32]. However, almost 50% of the sequences identified in our subtraction library have not been reported in As related expression studies in other plant species. Important among them, were the serine palmitoyl transferase (SPT), adenylyl phosphosulfokinase (APSK), ChaC, MATE, pathogenesis and defense related proteins (PDR), transport protein particle component Bet3p like (TRAPP beta3) involved in vesicular trafficking and the proteins with unknown functions. Based on the sequence identity and their functions in various metabolic pathways, we clustered the transcripts into different functional groups as discussed below.

### Oxidative stress and antioxidants

**Figure 4:**
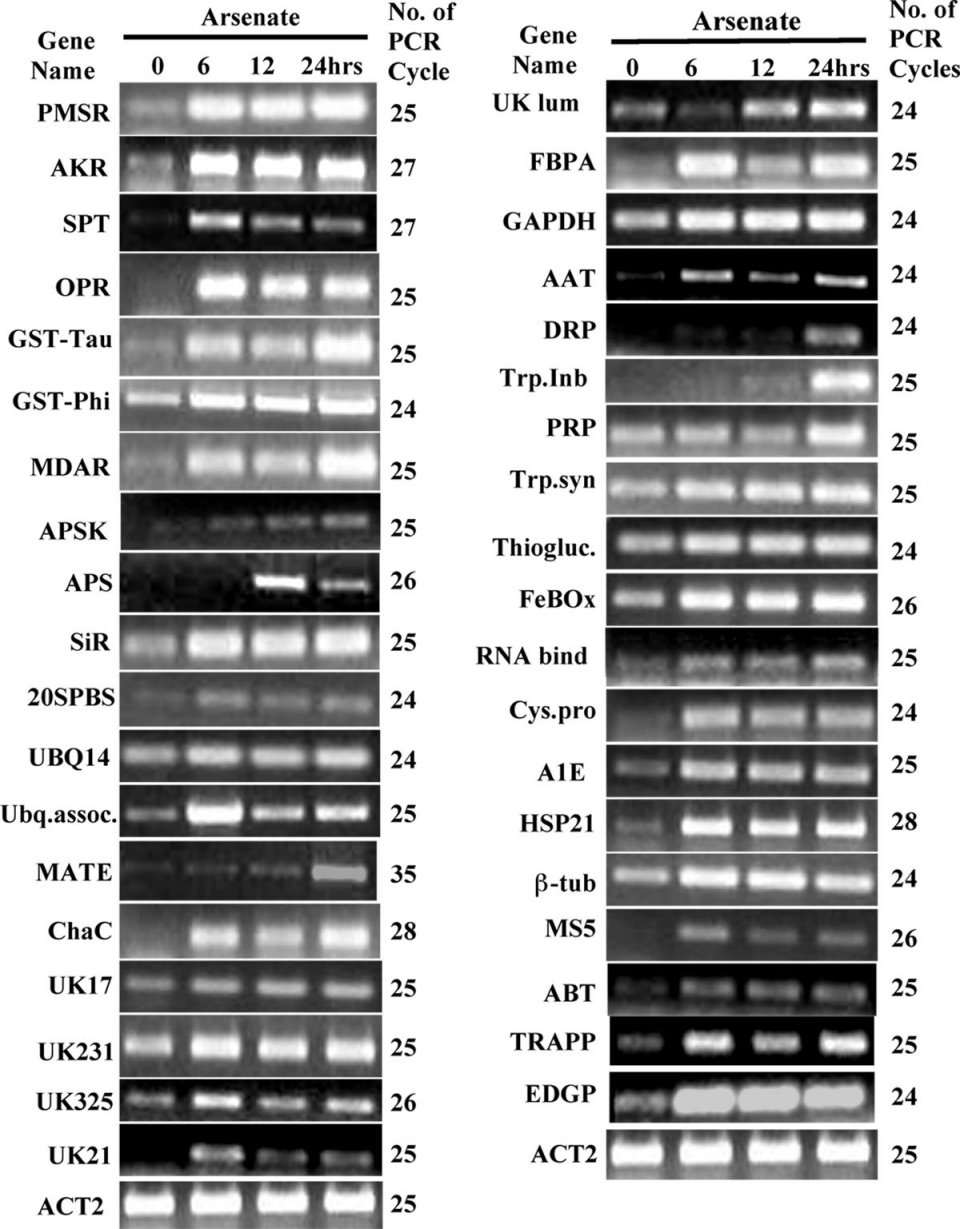
**Semi-quantitative RT-PCR analysis of the *C. abyssinica *transcripts corresponding to the subtracted cDNAs after 6, 12 and 24 hrs of arsenate treatments**. The details about the subtracted cDNAsequences identified by their serial numbers are given in Table 1. The number of optimized PCR cycles used for amplification for each cDNA clone is written on the right hand side of the panel for each cDNA. Actin2 gene, *ACT2*, was used as an internal control for equal loading of cDNA template of each clone.

Proteins involved in oxidative stress were highly represented in our subtracted cDNA library. There were four transcripts with 75% identity to the known aldo/keto reductases (AKR) and five transcripts similar to peptide methionine sulfoxide reductases (PMSR). AKR and PMSR have established roles in mitigating oxidative protein damage. Arabidopsis plants over-expressing PMSR3 were tolerant to methyl viologen, ozone, and metals such as Zn and Mn, illustrating its role as a vital antioxidant [33]. Recently, members of the NADPH dependent AKR family have been portrayed as antioxidants as well as cellular detoxification enzymes [34]. Arsenic has been shown to interact with phospholipid bilayers and thus modifying the properties of biomembranes by altering membrane fluidity [35]. Serine palmitoyl transferase (SPT), which catalyses the initial step of sphingolipid biosynthesis was present in our library, and was upregulated by AsV (Figure 4) and ozone treatments (Table 1 and 2). The lethal phenotype of SPT knockouts and reduced growth of Arabidopsis lines with RNAi downregulated SPT, illustrates the vital role of these molecules in membrane integrity [36].

The jasmonic acid signaling pathway is thought to mediate biotic as well as abiotic stress signaling. The 12-oxophytodienoate (OPDA) with structural resemblance to jasmonate is the first metabolic intermediate in the jasmonate synthesis pathway. It has been shown that the OPDA was capable of inducing the OPR, several GSTs and genes containing xenobiotic responsive elements [37]. Our subtracted library contains multiple copies of OPDA reductase (OPR), which was upregulated both in arsenate (Table 1, Figure 4) and ozone treatment (Table 2). Recently, members of the OPR family (OPR1, OPR2, and OPR3) have been shown to reduce the xenobiotic 2,4,6-trinitrotoluene (TNT) in Arabidopsis [38]. These studies and the upregulation of OPR in response to AsV exposure in crambe suggest the significance of OPRs in signaling and detoxification of toxic pollutants.

### Glutathione-S-Transferases and sulfate assimilation

Transcripts belonging to the glutathione-S-transferases (GSTs) form the largest group in the subtracted cDNA library. Most of these transcripts (12%) fall in the *Tau *subfamily (GST-*Tau*), while the remaining sequences (4%) are similar to the *Phi *subfamily. GSTs are considered to be involved in detoxification of both endogenous and xenobiotic compounds with electrophilic centers by the nucleophilic addition of glutathione [39]. Members of the ABC transporter family, human multidrug resistant proteins (MRPs) and yeast cadmium factor1 (YCF1), were found to export glutathione conjugated AsIII out of the cytoplasm [40,41]. However, GST mediated conjugation of glutathione with inorganic ions has not been proven. GSTs might be mitigating oxidative stress by other means as ectopic expression of tomato GST significantly improved the tolerance of yeast to H_2_O_2 _induced oxidative stress [42].

Another sequence present in our library, monodehydroascorbate reductase (MDAR) participates in the glutathione-ascorbate redox cycle that is responsible for the active oxygen species (AOS) scavenging process [43]. Ascorbate reacts with AOS and produces an unstable compound, monodehydroascorbate (MDHA) that dissociates into ascorbate and dehydroascorbate (DHA). The later is eventually reduced to ascorbate by DHA reductase using reduced glutathione as the source of electrons [43]. However, the increased regeneration of ascorbate directly from MDHA due to higher levels of MDAR could decrease the demand for glutathione [44]. It has been shown that tobacco seedlings overexpressing a tobacco GST exhibited a concomitant increase in the expression of MDAR, possibly due to a lower amount of reduced glutathione [44]. Surprisingly, both the GST-*Tau *and MDAR in our subtracted library displayed identical time-dependent expression patterns in response to arsenate (Figure 4), which is in agreement with a possible role of diminishing cellular concentration of GSH on induction of MDAR.

Our library also contains genes related to biosynthesis of cysteine, a precursor of glutathione biosynthesis, thus emphasizing the role of GSH in arsenic detoxification. Cysteine biosynthesis represents the reductive sulfate assimilation pathway. This is initiated by adenylylation of sulfate by sulfate adenylyl transferase or adenosine phosphosulfurylase (APS) followed by its reduction to sulfite by APS reductase (APR) and then to sulfide by sulfite reductase (SiR). The sulfide is incorporated into O-acetyl serine by cysteine synthase [45]. Considering the occurrence of elevated levels of GSH in plants overexpressing cysteine synthase [46], the presence of highly upregulated SiR and APS in our library prompted us to speculate that there is an overall increase in glutathione synthesis under AsV stress. Further studies are needed to establish a clear role for these enzymes in As metabolism.

### Ubiquitin-proteasome pathway

Genes involved in the proteasomal degradation system constitutes 8% of the subtracted cDNAs in our library. This includes the proteolytic beta-subunit of the 20S particle, polyubiquitin 14 (UBQ14) and an ubiquitin-associated protein. The elevated expression of 20S proteasome subunits and UBQ14 indicated that, most likely, the oxidized or the arsenite bound proteins in AsV stressed plant cells were ubiquitinated for proteasome-mediated degradation. Besides, the ubiquitin-associated protein present in our subtraction library contained a zinc ion-binding domain, which could be a crambe homologue of an Ubrp1 type of ubiquitin ligase (E3) that transfers the activated ubiquitin to target proteins [47]. Therefore, we suggest that the proteasome-mediated protein degradation machinery is vital to counteract the toxicity induced by As. However, further studies are needed to elucidate the exact roles of the genes involved in the proteasomal degradation system in As metabolism and detoxification.

### Transporters

Two membrane transporters, a multidrug and toxin extrusion pump (MATE) and a putative cation transporter associated protein (ChaC), were also found in the pool of our subtracted cDNA clones. MATE belongs to a large family of secondary transporters with 56 ORFs in the *A. thaliana *genome. An Arabidopsis MATE transporter, AtDTX1, was reported to transport alkaloids, antibiotics and other toxic compounds [48]. In addition, ectopic expression of AtDTX1 conferred Cd resistance to bacterial cells and two members of this family were upregulated in As treated rice seedlings [22]. In prokaryotes, the putative transporter regulatory protein ChaC is thought to be associated with a Ca^2+^/H^+ ^cation transporter, ChaA. A ChaC homologue from rice, OsARP, conferred salt tolerance when overexpressed in tobacco [49], suggesting a role for ChaC in abiotic stress tolerance. These transporters have not yet been characterized in plants and further studies are needed to understand their role in arsenic metabolism.

### Unknown novel proteins

There were five subtracted cDNA sequences that did not match to any of the characterized proteins in the database. However, they have homologs in Arabidopsis with very high sequence similarity, but with unknown function, and these transcripts were named as UK (unknown) with their colony ID. For example, UK325, one of the novel sequences, has a methyl transferase and a Suppressor of variegation-Enhancer of zeste-Trithorax (SET) domains for transferring a methyl group to its substrates from S-adenosyl methionine. Previously characterized arsenic methyl transferases in rabbit liver demonstrated a GSH-dependent transfer of methyl group from S-adenosyl methionine to As [50]. Further characterization of these unknown novel sequences will shed light on their role in As metabolism.

### Validation of PCR-Select subtraction data by RT-PCR

In order to confirm the upregulation of the subtracted cDNA clones in response to AsV exposure, a semi-quantitative RT-PCR was performed for each gene present in the subtracted cDNA library. Nearly 85% of the transcripts present in our library responded within 6 hrs after the AsV treatment (Figure 4). Both AKR and PMSR proteins were upregulated within 6 hrs after the As treatment and showed a similar expression pattern up to 24 hrs. MDAR and OPR responded within 6 hrs of AsV treatment and transcripts of MDAR continued to increase several folds higher at 24 hrs (Figure 4). A similar pattern of transcriptional response was observed in the case of SPT, known to participate in the ROS response. Transcripts of both subfamilies of GSTs were upregulated within 6 hrs of AsV treatment and the mRNA levels remained high throughout the period of As treatment.

Among the enzymes in sulfate assimilation, SiR showed strong induction throughout the duration of AsV treatments. The transcripts level of APSK increased consistently, however, APS was upregulated only at 12 hrs and then decreased after 24 hrs. Since the energetics of the activation of sulfate favors the reverse reaction [45], a delay in the induction of APS was expected as compared to the downstream enzymes in sulfate assimilation. The UBQ14 and 20S beta-subunit were significantly upregulated at 6 hrs and this higher expression level remained unaltered at all the time points tested. The expression pattern of the ubiquitin-associated protein was noticeably different, however, with a sharp increase in mRNA levels at 6 hrs and then decreased at the following time intervals. The secondary transporter, MATE, was induced in As treated seedlings only after 24 hrs of exposure to AsV, whereas, ChaC was induced at 6 hrs and continued the higher level of expression at 12 and 24 hrs. The unknown sequences, UK17 and UK231 were consistently upregulated at 6, 12 and 24 hrs after AsV treatment, whereas, UK21 and UK325 showed maximum mRNA levels at 6 hrs and then decreased steadily over time. The unknown protein with a domain similar to the luminal binding protein (UK-lum) was upregulated only after 12 hrs with further increase in expression at 24 hrs.

The expression levels of the sequences corresponding to the glycoprotein EDGP, FeBOx, HSP21, β-tubulin, and TRAPP beta3 were upregulated and maintained many fold higher expression level at 6, 12, and 24 hrs after AsV treatment. A similar pattern but at lower expression levels was observed in case of thioglucosidase, tryptophan synthase, cysteine proteinase, and aldose epimerase. Contrary to this trend, both trypsin inhibitor and defense related proteins were strongly induced only after 24 hrs of AsV exposure. The differential regulation of most of these subtracted cDNA suggests their role in As metabolism and detoxification processes.

### Comparison with phosphate and ozone stress

**Figure 5:**
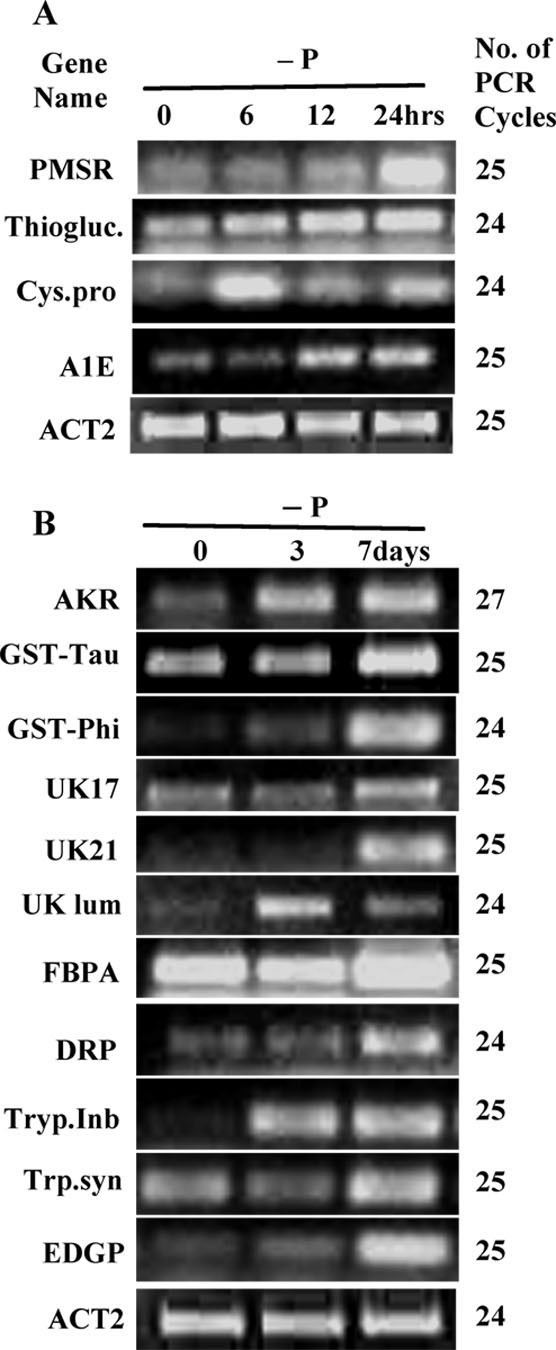
**Semi-quantitative RT-PCR analysis of the *C. abyssinica *cDNAs in response to phosphate deficiency**. Phosphate deficiency at 6, 12 and 24 hrs for short term (**A**), and 3 and 7 days for long-term exposure (**B**). Sequences are identified by their serial numbers as shown in Table 1. The number of optimized PCR cycles used for amplification for each cDNA clone is written on the right hand side of the panel for each cDNA. Actin2 gene, *ACT2*, was used as an internal control for equal loading of cDNA template of each clone.

A comparison of the expression of the homologous transcripts in Arabidopsis in response to phosphate deficiency and ozone-induced oxidative stress revealed an overlapping effect of these related stress conditions (Table 2). Expression levels of one third of the sequences in the subtracted cDNA library were affected by varying phosphate level in the media and strikingly, all of them were found to have higher mRNA levels in phosphorus deficiency. This trend was verified in crambe by semi-quantitative RT-PCR in seedlings grown with or without phosphate for 6, 12, and 24 hrs as short-term and 3 and 7 days as long-term exposure. In crambe, only a few transcripts such as cysteine proteinase, thioglucosidase, aldose epimerase, and PMSR showed increased transcription within 24 hrs of phosphate deficiency (Figure 5). However, the transcripts encoding EDGP, AKR, trypsin inhibitor, FBPA, GSTs, UK17, tryptophan synthase, defense related protein, UK-lum, and UK21 were upregulated after 7 days of phosphorus deficiency.

**Table 2:** Arabidopsis sequences homologous to arsenate-induced subtracted cDNAs from *C. abyssinica *showing differential expression in response to low phosphate (LP) levels and oxidative stress with ozone from microarray experiment analysis http://www.ebi.ac.uk/microarray-as/aer/#ae-main[0]

**Description**	**Relative transcript levels in plants with LP**	**Transcript levels in plants with Ozone treatment**
Peptide methionine sulfoxide reductase (PMSR)	NS	Upregulated *
Serine palmitoyl transferase (SPT)	NS	Upregulated *
Oxophyto dienoate reductase (OPR)	NS	Upregulated **
Glutathione-S-Transferase *Tau *subfamily (GST-Tau)	Higher in LP *	Upregulated **
Glutathione S Transferase *Phi *subfamily (GST-Phi)	Higher in LP **	Upregulated **
Sulfate adenylyltransferase (APS)	NS	Upregulated **
20S proteasome beta subunit (20SPBS)	NS	Upregulated **
MATE - family drug transporter (MATE)	Higher in LP **	Upregulated **
Unknown (UK17)	Higher in LP **	NS
Unknown (UK325)	Higher in LP **	Downregulated *
Unknown (UK21)	Higher in LP **	Upregulated **
Aspartate amino transferase (AAT)	Higher in LP **	NS
Defense related protein (DRP)	Higher in LP **	Upregulated **
Trypsin inhibitor (Tryp.Inb)	Higher in LP **	Upregulated **
Pathogenesis related protein (PRP)	Higher in LP **	NS
Tryptophan synthase (Trp.syn)	Higher in LP **	Upregulated *
Thioglucosidase precursor (Thiogluc.)	NS	Downregulated **
Iron ion binding oxidoreductase (FeBOx)	Higher in LP *	Downregulated **
RNA-binding domain containing protein (RNA bind.)	Higher in LP **	NS
Cystein proteinase (Cys.pro)	Higher in LP **	NS
Aldose 1 Epimerase (A1E)	Higher in LP **	Upregulated **
Beta tubulin (β- tub.)	Higher in LP *	NS
Male sterility family protein (MS5)	Higher in LP *	NS
Anthranilate benzoyl transferase (ABT)	Higher in LP *	Upregulated **
Extracellular dermal glycoprotein (EDGP)	Higher in LP *	Upregulated **

LP = Low Phosphate NS = Non significant ** Significant at P = 0.01 *Significant at P = 0.05

Ozone is known to induce oxidative stress and transcripts encoding enzyme with antioxidant property were upregulated in response to ozone stress. One-third of the transcripts related to the subtracted cDNAs in our library showed elevated expression levels when Arabidopsis seedlings were subjected to oxidative stress by ozone (Table 2). This supported the previously reported observations of As-induced ROS resulting in lipid peroxidation and higher SOD activity in *Holcus lanatus *[51].

## Conclusions

Present study proves that crambe plants have the potential for As phytoremediation because they are highly tolerant to As and in addition can accumulate high levels of this toxic metal in the above ground parts. Moreover, our study identifies several genes that are highly upregulated in response to AsV stress and that encode novel proteins and proteins involved in various vital metabolic pathways. These arsenate-induced proteins may play a crucial role in As metabolism in plants. By further characterizing these differentially expressed genes, we may be able to acquire knowledge essential for developing novel strategies for efficient phytoremediation as well as engineering As tolerant crop plants with reduced translocation of this deadly metalloid to the edible parts of plants.

## Methods

### Plant growth and arsenate treatments

*Crambe abyssinica *(cv. BelAnn) seeds were surface disinfected using 70% ethanol for five minutes followed by a treatment with 30% clorox for 30 minutes. Seeds were washed four times with sterile deionized water and then germinated on 0.5 × MS (Murashige and Skoog) agar media. Germinated seeds were aseptically transferred to 0.5 × MS liquid media in 250 ml flasks and grown under controlled conditions (16/8 hour light/dark periods at 22°C) with constant shaking. After 10 days, sodium arsenate (Na_2_HAsO_4_.7H_2_O) at various concentrations (0, 100, 150, 200, 250, 300 and 350 μM) was added to the medium and plants were grown for an additional 7 days to determine the optimum concentration for As treatment. A final concentration of 250 μM AsV, where plants showed significant reduction in biomass without exhibiting severe toxicity symptoms, was then used for treating plants for cDNA subtraction experiments. Ten days old seedlings were exposed to AsV for 24 hrs and the seedlings were harvested, frozen in liquid nitrogen, ground in to a powder and then stored at -80°C for further processing. For inducing the phosphate (P) deficiency, plants were grown in 0.5 × MS phosphate dropout liquid media (pH 7.0) in 250 ml flasks under 16/8 hour light/dark periods at 22°C with constant shaking. After the various time periods (6, 12, and 24 hrs as short-term and 3 and 7 days as long-term exposure), seedling samples were harvested, washed in deionized water and stored at -80°C till further use.

In order to compare the As accumulation in different Brassica species (*Brassica napus*, *B. compestris *cv Turkey, *B. compestris *cv Yellow Sarson, *B. oleracea*, *B. rapa*) and *C. abyssinica*, the seeds were germinated and grown for 14 days on 0.5 × MS agar media supplemented with various concentrations (0, 200 and 400 μM) of sodium arsenate. Total As in the shoot tissues was analyzed using Inductively Coupled Plasma-Mass Spectroscopy (ICP-MS).

### RNA isolation, cDNA synthesis and PCR-Select subtraction

Total RNA was extracted from control and AsV treated samples using RNeasy Plant Mini kit (Qiagen) followed by the mRNA isolation using NucleoTrap mRNA isolation kit. Double stranded cDNA synthesis and cDNA subtractions were performed using the PCR select cDNA subtraction kit (Clontech, CA) according to the manufacturer's protocol. In short, the cDNAs from driver (control) and tester (As-treated) samples were digested with endonuclease *Rsa*I and the tester cDNAs were ligated to two different adapters at either end of the cDNAs separately [52]. The twin samples of tester differing in the adapters were denatured and allowed to hybridize in separate tubes with excess denatured driver cDNAs in each tube. Apart from enriching the differentially expressed cDNAs, this step normalized the high and low abundant sequences. The tester samples were then mixed without denaturation in presence of the fresh denatured driver cDNAs and allowed to anneal overnight. Subsequent PCR amplifications, first with primers corresponding to both adapters then with nested primers, selectively amplified and enriched the differentially expressed cDNAs. A reverse subtraction, by switching the tester and driver cDNAs was also performed for the purpose of differential screening. In order to construct the subtracted cDNA library, the subtracted and PCR amplified products were ligated to the pGEM-T-Easy T/A cloning vector (Promega) and transformed into DH5α cells.

### Differential screening

A differential screening procedure was used to eliminate the false positives in the subtracted cDNA library. This step was carried out using a differential screening kit (Clontech) following the manufacturer's protocol. In this procedure, the subtracted cDNAs blotted onto the nylon membranes were hybridized with forward and reverse subtracted probes and the signals were compared for each clone. Colonies containing plasmids with cloned cDNAs were grown in liquid LB medium overnight and an equal amount of each culture was spotted in duplicate on two replicate nylon membranes, which were placed on LB plates and incubated at 37°C overnight. Membranes containing the colonies were transferred to Whatmann filters pre-saturated with denaturing solution (0.5 M NaOH, 1.5 M NaCl), and then to Whatmann filters pre-saturated with neutralizing solution (0.5 M Tris-HCl, 1.5 M NaCl). Each membrane was air-dried and the DNA was fixed by baking the membranes at 80°C for 2 hours. These membranes were then hybridized with either ^32^P labeled forward or reverse subtracted probes overnight as described previously [53] and washed with low stringency (2 × SSC, 0.5% SDS) and high stringency (0.2 × SSC, 0.5% SDS) wash buffer before exposing to X-ray film at -80°C overnight. The hybridization signal strength for each cDNA clone on membrane probed with forward subtracted cDNA was compared with cDNA clones on the duplicate membrane probed with reverse subtracted cDNA.

### DNA sequencing and gene identification

A total of 105 differentially expressed cDNA clones were sequenced both in forward and reverse orientations. The DNA sequences of these clones were used to search the NCBI Genebank database to identify homologues sequences and their identity using the BLAST algorithm [54]. All the subtracted cDNA sequences were used for BLAST search against both DNA and protein sequences on the database and their homologous gene sequences were identified both from Prokaryotes and Eukaryotes.

### Semi-quantitative RT-PCR

For RT-PCR, 10 days old crambe seedlings were treated with 250 μM sodium arsenate and the samples were harvested after 6, 12 or 24 hours treatment. Total RNA was extracted using the RNAeasy Plant mini kit (Qiagen), and cDNA synthesized from total RNA using Thermoscript RT-PCR kit (Invitrogen) following the manufacturers protocol. Primers for each subtracted cDNA were designed using Primer3 [55]. Threshold of the number of cycles was determined by an optimization step in which the PCR was performed from a known amount of cDNA for a range of cycles. The number of PCR cycles selected for the experiments were chosen from the plot where the trend line exhibited highest correlation to exponential amplification. Once the number of cycles for a few genes was optimized, it was extrapolated to all other genes in the library based on their microarray expression (ArrayExpress) values, as it represented the relative expression level of each mRNA. In addition, the constitutively expressed actin2 gene, *ACT2*, was used as an internal control to verify that an equal amount of cDNA template was used in each PCR reaction. A touchdown PCR was performed for all the transcripts with annealing temperature varying from 64°C to 52°C. Similarly, to examine the effect of phosphate deficiency on gene expression, seedlings were grown in half strength MS medium with or without phosphate and after 6, 12 or 24 hrs treatment samples were collected. In order to evaluate the long-term effects of phosphate deficiency, RNA was isolated from seedlings grown in phosphate deficient media for 3 and 7 days.

### Statistical analysis and comparison with microarray data

All statistical analyses were performed using SAS software (SAS version 9.1). For comparison, Arabidopsis microarray data on phosphate [[56], ArrayExpress ID: E-MEXP-791] and ozone treatments [[57], ArrayExpress ID: E-MEXP-342], were downloaded from the ArrayExpress [58], processed by Bioconductor in R programming environment and annotated using a Perl script. ANOVA was performed on the log-transformed expression values for genes which were present in the subtracted library (25 transcripts in Affymetrix array) identified by AGI locus and followed by Dunnett's procedure [59] for ozone treatments. Duncan's New Multiple range test [60] was performed for the pair-wise comparison of expression levels in high phosphate and low phosphate treatments.

## Authors' contributions

BP performed the subtraction procedure, cDNA cloning, sequence analysis, RT-PCR, analyzed data and participated in writing the manuscript. SK grew crambe and Brassica species in arsenic media, performed the arsenic uptake assays and analyzed the data. OPD designed and supervised this whole project and participated in writing the manuscript. All authors read and approved this manuscript.

## Authors' Information

OPD is an Assistant Professor and BP is a PhD candidate in the Department of Plant, Soil, and Insect Sciences, University of Massachusetts Amherst. SK is currently an undergraduate student at Cornell University.
